# A strategy using NMR peptide structures of thromboxane A2 receptor as templates to construct ligand-recognition pocket of prostacyclin receptor

**DOI:** 10.1186/1471-2091-6-23

**Published:** 2005-11-04

**Authors:** Cheng-Huai Ruan, Jaixin Wu, Ke-He Ruan

**Affiliations:** 1 From the Vascular Biology Research Center and Division of Hematology, Department of Internal Medicine, The University of Texas Health Science Center, Houston, 6431 Fannin St., Houston, Texas 77030, USA

**Keywords:** G protein-coupled receptor, GPCR, prostacyclin (prostaglandin I_2 _(PGI_2_) receptor, protein modeling, thromboxane A_2 _receptor, NMR structure, synthetic peptide.

## Abstract

**Background:**

Prostacyclin receptor (IP) and thromboxane A2 receptor (TP) belong to rhodopsin-type G protein-coupling receptors and respectively bind to prostacyclin and thromboxane A2 derived from arachidonic acid. Recently, we have determined the extracellular loop (eLP) structures of the human TP receptor by 2-D 1H NMR spectroscopy using constrained peptides mimicking the individual eLP segments. The studies have identified the segment along with several residues in the eLP domains important to ligand recognition, as well as proposed a ligand recognition pocket for the TP receptor.

**Results:**

The IP receptor shares a similar primary structure in the eLPs with those of the TP receptor. Forty percent residues in the second eLPs of the receptors are identical, which is the major region involved in forming the ligand recognition pocket in the TP receptor. Based on the high homology score, the eLP domains of the IP receptor were constructed by the homology modeling approach using the NMR structures of the TP eLPs as templates, and then configured to the seven transmembrane (TM) domains model constructed using the crystal structure of the bovine rhodopsin as a template. A NMR structure of iloprost was docked into the modeled IP ligand recognition pocket. After dynamic studies, the segments and residues involved in the IP ligand recognition were proposed. A key residue, Arg173 involved in the ligand recognition for the IP receptor, as predicted from the modeling, was confirmed by site-directed mutagenesis.

**Conclusion:**

A 3-D model of the human IP receptor was constructed by homology modeling using the crystal structure of bovine rhodopsin TM domains and the NMR structures of the synthetic constrained peptides of the eLP domains of the TP receptor as templates. This strategy can be applied to molecular modeling and the prediction of ligand recognition pockets for other prostanoid receptors.

## Background

Prostanoids including thromboxane A_2 _(TXA_2_) and prostaglandins D_2 _(PGD_2_), E_2 _(PGE_2_), F_2 _(PGF_2_) and I_2 _(PGI_2_) act as local hormones in the vicinity of their production sites to regulate hemostasis and smooth muscle functions, which are mediated by specific prostanoid receptors in the plasma membrane. The receptors are classified into five basic types based on their cognate prostanoid (PGD_2_, PGE_2_, PGF_2_α, PGI_2 _or TXA_2_) and are termed DP, EP, FP, IP and TP receptors, respectively [1]. In addition, the EP receptors are subdivided into four subtypes (EP1, EP2, EP3 and EP4) based on their different signaling responses to PGE_2_. Human TP was first purified from a platelet in 1989 and its cDNA was cloned from placenta in 1991 [2,3]. Other prostanoid receptor cDNAs, including those for DP, EP1, EP2, EP3, EP4, FP and IP have also been cloned, and their primary biologic functions have been identified.

Prostanoid receptors can be divided into two functional groups using smooth muscle assays: relaxant receptors (including DP, EP2, EP4, and IP) and excitatory receptors (including EP1, FP, and TP). All known prostanoid receptors belong to the rhodopsin-type G protein-coupled receptor (GPCR) super-family, which has seven conserved TM domains. The diverse and/or opposite biological functions of the individual prostanoid receptors involve selective ligand binding on their extracellular and/or TM domains and the interaction with different heterotrimeric G proteins on their intracellular domains [1].

For over a decade, structural and functional studies of prostanoid receptors have focused on the identification of the specific sites for ligand binding and G protein coupling. The homology alignment-based mutagenesis studies of the receptor TM domains have suggested that the conserved regions in the third and seventh TM domains are involved in binding the core structures of the prostanoids, which consist of a carboxylic acid, a hydroxyl group on carbon 15 and two aliphatic side chains [4-6]. However, the TM domains belong to the conserved regions in all known prostanoid receptors, and therefore, probably do not define the differences among the receptors in specific interactions with their ligands. Current focus has been directed toward the involvement of the extracellular loops (eLPs) of prostanoid receptors in the selective ligand recognition, similar to some other GPCRs [7-15]. However, interpretation of the eLP specific determinants response to the specific ligand binding among prostanoid receptors is limited by the lack of experimental 3-D structural information for the extramembrane domains. Structural characterization of both the extracellular and intracellular domains of the prostanoid receptors needed to elucidate the molecular mechanisms of specific ligand recognitions and G protein couplings remains a major challenge.

Molecular modeling has been widely used to create a working model of the GPCR using a known x-ray crystal structure of rhodopsin. Electron diffraction and electron microscopy studies have succeeded in determining the structure of the GPCR-like protein, bacteriorhodopsin (BR), at low and medium resolution [16,17]. The high-resolution 3-D structure of BR has also been determined by X-ray crystal studies [18,19]. Using the BR structure as a template, a number of working models of the conserved TM domains for the rhodopsin-type GPCRs have been constructed by homology modeling [6,20-32]. Most of these models assumed that the GPCRs had the same spatial arrangement for the seven helices as BR. However, it has recently been suggested that BR is not a suitable template for the construction of GPCR TM models because BR does not function as a GPCR and there is no overall significant sequence similarity between BR and the GPCRs [33]. Further evidence is given from the structural maps of bovine, frog and squid rhodopsins [34-39], which indicate that the arrangement of the helices in the rhodopsins is indeed different from that in BR. As such, it is believed that the crystal structure of the TM domains of bovine rhodopsin (bRH) is a more suitable template for constructing working models of mammalian GPCR TM domains, which include the prostanoid receptors.

It is almost impossible to build the structural extra- and intra-cellular loop models for the prostanoid receptors using the corresponding structures of BR or bRH due to a number of differences, including size and sequence variation. However, understanding the structural features of the extracellular loops of the prostanoid receptors is the key step toward uncovering the ligand binding selectivity. To overcome the difficulty recently, we have successfully determined the solution structures of the individual eLP domains for the human TP receptor, and identified that its ligand (antagonist) recognition pocket is mainly formed by the second eLP (eLP_2_) and the disulfide bond between the eLP_2 _and the first eLP (eLP_1_). This was done using newly-developed concepts including, computation-guided constrained peptide synthesis and NMR experiment-guided mutagenesis approaches. These results offer a basis for understanding the ligand recognition sites for the other prostanoid receptors through the homology modeling approach using the NMR structures of the eLP segments, which share many similarities with the other prostanoid receptors, especially the IP receptor. In this paper, we describe the approaches used to construct the working model for the IP receptor by homology modeling using x-ray TM structures of bRH and NMR structures of the eLPs of the TP receptor as templates. We also discuss its value in predicting the residues involved in ligand recognition for the IP receptor through modeling and mutagenesis.

## Results

### Homology modeling for the seven TM domains of the human IP and TP receptors using the crystal structure of the TM domains of the BR as a template

**Figure 1:**
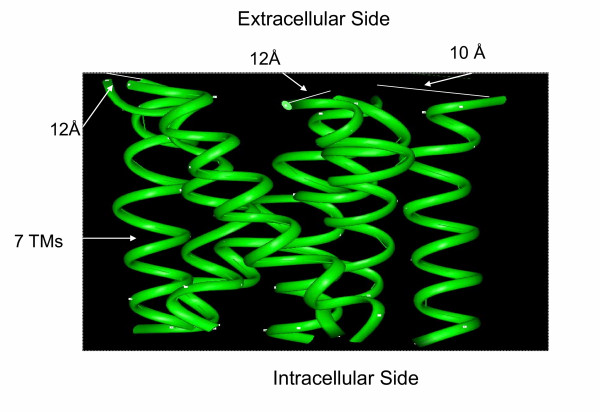
Homology modeling of the seven TM domains of human IP receptor. 3-D backbone structure of the seven TM domains of the human TP receptor were created by homology modeling using the crystal structural backbones of the TM domains of the bovine rhodopsin as templates.

The amino acid sequences of the human IP and TP receptors and bRH were downloaded from Gene Bank. The first step is to highlight the multiple sequence alignments between the TM domains of bRH, TP, and IP receptors. The putative TM domains of the IP and TP receptors are based on the hydropathy analysis described in the original cloning papers [3,40]. The significant similarities (data not shown) in the TM domains reflect the similarity of the general backbone structures between the TP and IP receptors with bRH. To construct the homology models of the TM domains of the IP and TP receptors, the crystal structures of the TM domains of bRH were used as a template, and its backbone conformations were adopted for the each TM domains of the IP and TP receptors using the Insight II package on a SGI Octane workstation. The well-developed commercial software, Insight II, has been widely used in various academic fields for protein modeling. The detailed steps have been described previously [41,42]. The backbone structural model of the TM domains of the IP (Figure 1) and TP (data not shown) were obtained. It shall be indicated that the gaps between the template and objects were fixed by the molecular annealing computation.

### Structural features of the reported 3-D NMR structures of the eLPs of the TP receptor

Synthetic peptides corresponding to the eLP domains of the human TP receptor were mimicked by a constrained peptide technique with the N- and C-termini connected by a homocysteine disulfide bond [43,44]. The constrained eLP_2 _peptide was likely able to adopt an active conformation, which was evidenced by the binding of the peptide to the receptor ligand using fluorescence and CD spectroscopic studies [43-45]. Recently, by way of 2-D ^1^H NMR experiments, complete ^1^H NMR assignments for the 2-D spectra including, nuclear Overhauser effect correlation spectroscopy (NOESY), total correlation spectroscopy (TOCSY), double-quantum-filtered correlation spectroscopy (DQF-COSY), and structural construction were used to determine the overall 3-D structures of the constrained peptides mimicking the three TP eLPs (eLP1, [43], eLP2 [44] and eLP3 [45]). All of the NMR structures indicate the presence of β-turns in the loops [43]. The distance between the N- and C-termini of the peptides shown in the NMR structures was 10–14.2 Å, which matched the distance between the two TM helices connecting the eLPs in the TM domain model of the TP receptor. These structural features allowed the NMR structures of the constrained TP eLP peptides to be grafted onto the region of the TP receptor model in a configuration without further modifications [43,45]. This study suggests that the approach using the constrained loop peptide greatly increases the likelihood of characterization of the structural features of the extracellular domains of the TP receptor. It offers a structural template to model the extracellular domains of other prostanoid receptors, which as of yet have no defined crystallographic structures. The IP receptor is one example which shares significant similarities in its extracellular domains with those of the TP receptors.

### Sequence alignment of the three eLPs between the human IP and TP receptors

**Figure 2:**
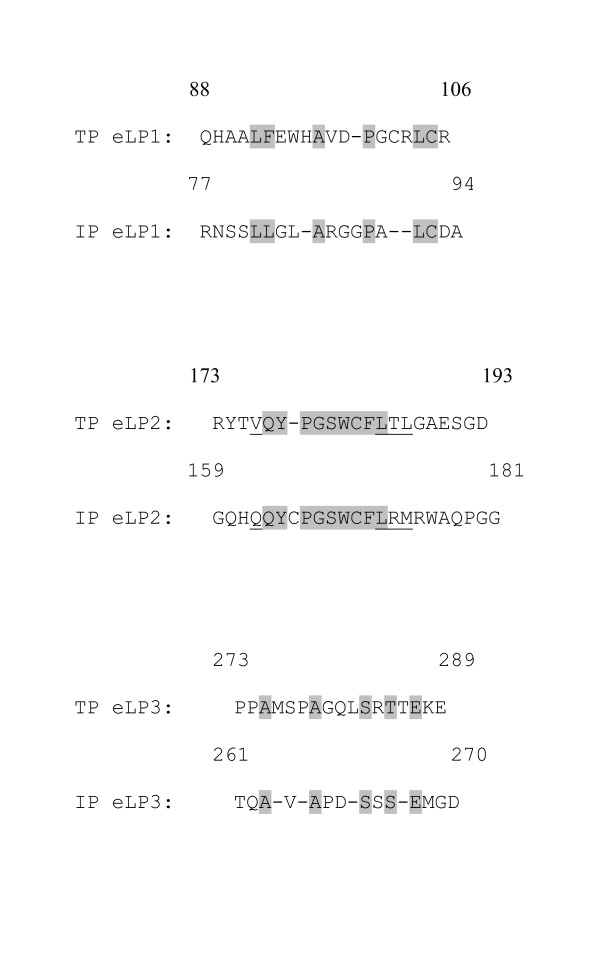
Sequence alignment of the eLPs between the human IP and TP receptors. The sequences of the putative eLP domains of the human IP (45) and TP (3) receptors were aligned by a sequence alignment program in Insight II software package and manual adjustment. The identical and highly similar residues between the TP and IP are shaded. The residues in the eLP_2 _regions previously identified important to the ligand binding for the TP receptor (10) and their corresponding residues in the IP receptor are underlined.

Sequence analysis has indicated that the amino acid residues in the eLP domains are not conserved between bRH and prostanoid receptors, such as the IP receptor. This has limited the use of the crystal structure of the eLP domains of bRH for homology modeling of the prostanoid receptors. Thus, the experimental NMR eLP structures of the TP receptor become useful in constructing a model of the IP receptor because they are conserved in the eLP domains. The eLP domains of the IP and TP receptors were identified from the hydropathy analysis described by the original cloning papers [3,40]. The three eLPs between the TP and IP were aligned and are shown in the Figure 2. The lengths of the three IP eLP domains are similar to those of the TP receptor, which allows them to align with no major gaps. The Cys residues (Cys105 in the eLP_1 _and Cys183 in the eLP_2_) of the TP receptor that form a disulfide bond [43], thereby controlling the conformation of the eLPs, are conserved in the IP receptor (Figure 2). Among the three eLPs, the most conserved region is localized in the eLP_2 _(40% identity, Figure 2), which has been identified to play a major structural role in forming the ligand recognition site for the TP receptor [10]. In addition, there are 33% and 36% of the sequence homologies for the eLP1 and eLP3, respectively (Figure 2). Thus, using the NMR structures of the TP eLPs as a template for modeling the IP eLPs will be useful in rationalizing the structural and functional features of the eLPs in the IP receptor. The individual similarities of the alignment are listed in the Figure 2.

### Homology modeling for the eLP domains of human IP receptor

**Figure 3:**
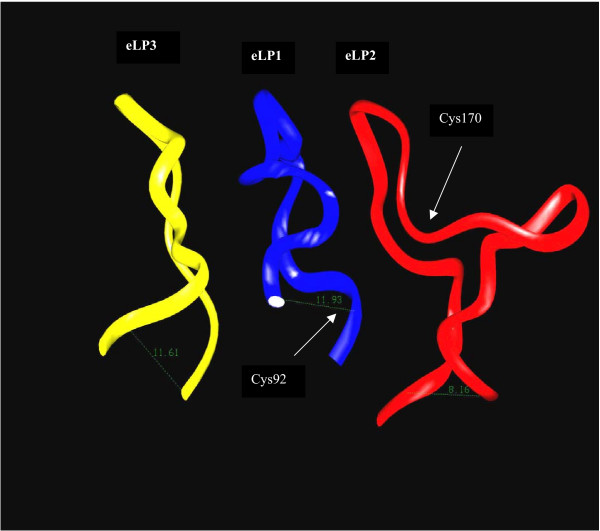
The predicted backbone structures of the three eLP domains of the human IP receptor. The 3-D structural models of the eLPs were constructed by homology modeling with the NMR structures of the eLP_2 _(48), the eLP3 (49) and the eLP1 (unpublished data) of the human TP receptor as templates using the molecular modeling package of Insight II and Discover software packages. The conformation of the eLP2 is placed in a position with respect to the formation of a disulfide bond between the Cys92 in the eLP1 and Cys170 in the eLP2.

**Figure 4:**
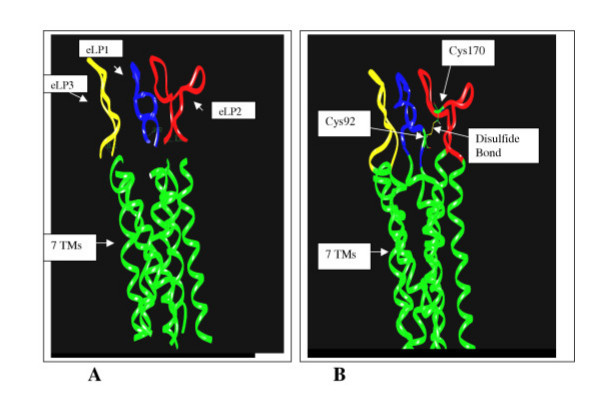
Configuration of the modeled 3-D backbone structures of the three IP eLP domains (Figure 4) onto the working model of the seven TM domains of the IP receptor. Before (A) and after (B) the connections of the eLP structures to the seven TMs through chemical bonds are displayed for their comparison.

The backbone structures of the individual IP eLPs were constructed by homology modeling using the NMR structures of the TP eLPs as templates with respect to the disulfide bond formation between Cys92 in the eLP_1 _and Cys170 in the eLP_2 _of the IP receptor (Figure 3). After energy minimization, each eLP structure was configured to the TM domain model. Each configuration was made based on the three following considerations: 1) placing the N- and C-termini of the eLP_1_, eLP_2 _and eLP_3 _to the C- and N-termini of the TM2 and TM3, TM4 and TM5, and TM6 and TM7; 2) matching the distances of the N- and C-termini of the eLPs with the corresponding distance between those of the TM helix (Figure 4A), 3) connecting the eLPs to the TMs through chemical bonds by Insight II calculation using Discover program (Figure 4B), and 4) forming a disulfide bond between Cys92, at the end of the eLP_1_, and Cys170, in the center position of the eLP_2 _(Figure 4B). After completing the configuration, 500 steps of energy minimization were used to refine the conformation of the model eLPs. As shown in Figure 4B, the constructed eLPs could be fitted into the conserved TM domains with respect to the cysteine disulfide bond between eLP_1 _and eLP_2_, without major violations in the structural calculation or significantly altering the original backbone structures in the original conformations before the connection (Figure 4A). Dynamic studies for the parent NMR structures of the TP eLPs have been described in our recent publication [43], in which 20 structural conformations have been generated and used to evaluate the loop folding. Limited conformational changes (rmsd = 1.2 Å) were observed [43]. The modeled structures of the IP eLPs described above adopted a similar variation of the conformations (data not shown). This information indicated that the folding of the IP eLPs is in a reasonable conformation.

### Ligand docking in the IP receptor model

To understand ligand selectivity and the action mechanisms of the IP receptor, it is very important to identify its specific ligand recognition/binding site. The IP receptor was cloned a decade ago, many attempts have been made to localize the segments and residues important to its ligand binding activities. However, little structural information is available. Through molecular modeling, we have made attempts to solve this problem.

**Figure 5:**
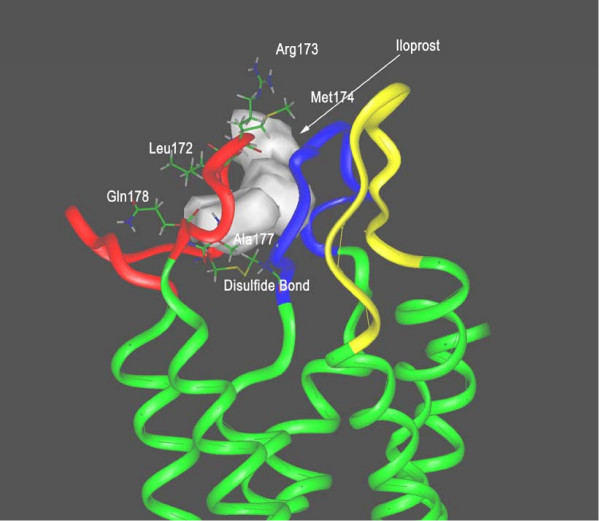
Ligand docking with the eLP domains of the IP receptor. The four residues including Gln162, Leu172, Arg173 and Met174 in the IP eLP2 (eLP1, bleu color; eLP2, red color and eLP3, yellow color) in contact with iloprost were predicted by the sequence alignment (Figure 1) using the identified four residues (Val176, Leu185, Thr186 and Leu187) in the TP eLP2 contacted with SQ29,548 (10) as a template. 3-D NMR structure of the IP receptor agonist, iloprost (42) was docked into the putative ligand recognition pocket formed by the three eLPs with respect to the contacts with Leu172, Arg1173 and Met174 in the opening of the pocket. In addition, the two residues, Ala177 and Gln178 involved in contacts with iloprost predicted by NMR spectroscopic studies (42) were also used as constraints for the iloprost docking to the recognition pocket. The configuration of the model was minimized using 1,000-step energy minimization after the iloprost was docked into the pocket. The TM domains of the IP receptor are showed with green colors.

The IP receptor ligand, prostacyclin, is not stable in solution and does not have 3D structural information. Recently, we have determined the solution structure of a stable IP receptor agonist, iloprost, using NMR spectroscopy [42]. It offers an experimental structure for molecular docking between the ligand and the IP receptor. On the other hand, based on the NMR experimental and mutagenesis results obtained for the TP receptor, the four residues (Val176, Leu185, Thr186 and Leu187) identified as being involved in ligand recognition are mainly localized in the eLP_2 _region [43]. The results indicated that perhaps the corresponding residues in the conserved IP eLP_2 _region are also likely to be involved in ligand recognition. This hypothesis has been supported by our recent NMR spectroscopic studies using a synthetic eLP_2 _fragment [42]. This information provided the basis for our study of the interaction between the IP receptor and its ligand using a molecular docking approach. The NMR structure of iloprost was docked into the pocket of the IP model corresponding to the ligand recognition pocket of the TP receptor as identified by NMR and mutagenesis studies [10] using Insight II and Discover computation procedures. The residues crucial to the TP ligand recognition [10] were used as a basis for localizing the possible residues involved in ligand contact in the IP receptor model (Figure 5). The orientation of the iloprost structure was positioned in contact with the side chains of the three of the four residues (Gln162, Leu172, Arg173 and Meth174, Figure 2 and 5) in the IP eLP2, corresponding to the previously identified contact sites among the residues of the TP eLP2 at Val176, Leu185, Thr186 and Leu187 with SQ29,548 (a TP receptor ligand) (10). In addition, it has been found that the residues Ala177 and Gln178 in the IP eLP2 fragment could interact with iloprost by our NMR spectroscopic studies [42]. It has also been taken for the consideration of the iloprost docking with the IP receptor (Figure 5). The structural complex of iloprost and the IP receptor was then subjected to energy minimization to find the best-fitting position of the iloprost conformation in the proposed ligand recognition site of the receptor (Figure 5). During the minimization, the main changes of the structures were the side chains of the loops, but no significant structural alternation for iloprost and the loop backbones were observed.

Recently, we have published the structural information of the ligand recognition site of TP receptor [43]. The disulfide bond involved in forming the ligand recognition pocket in the TP receptor has been confirmed by the reducing of the disulfide bond in the presence and absence of the ligand binds and the Cys mutations [43]. From this IP receptor model, we have also learned that the disulfide bond between the eLP_1 _and eLP_2 _is involved in forming the ligand recognition pocket (Figure 5). Reduction of the disulfide bond will alter the active conformation of the pocket of the receptor.

### Confirmation of the NMR experiment-based modeling for prediction of the residues involved in IP receptor ligand recognition using recombinant IP receptor

**Figure 6:**
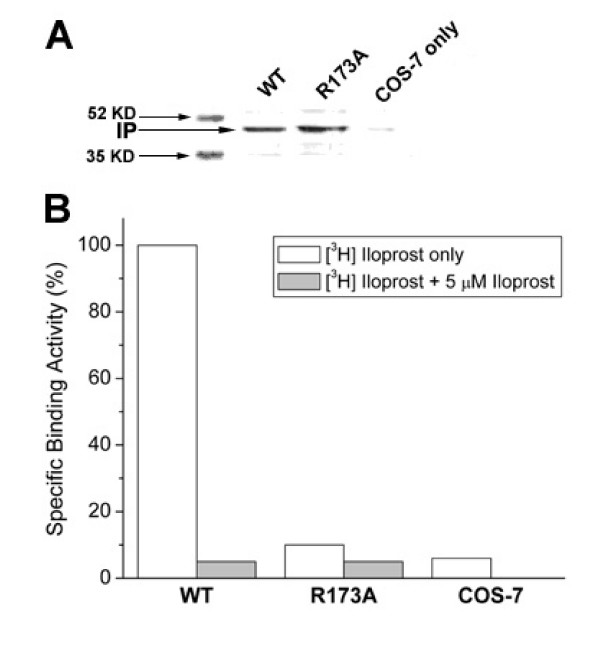
Analysis for the mutation of Arg173 to Ala residue of the recombinant human IP receptor. A). Western blot analysis. Fifty micrograms of COS-7 cells transfected with wild-type (WT) or a mutant IP receptor (R173A) cDNA was subjected to SDS-PAGE and transferred onto a nitrocellulose membrane. The membrane was probed with rabbit anti-IP peptide antibody. (B) The ligand binding activities of wild-type and mutant TP receptor. 300 μg of the protein prepared from the COS-7 cells transfected with cDNA of the wild-type (WT) or the R173A mutant was incubated with 4 nM [^3^H]-iloprost (30,000 cpm) in the absence or presence of unlabeled iloprost (1 μM) in a reaction volume of 100 μl. After 1 h incubation, the reaction was stopped and the binding activity of the recombinant IP receptor was measured as described in the methods. The binding activity of wild-type receptor was considered as 100% (2,000 cpm).

Distinct characteristics are noted among the four predicted residues important to ligand recognition for the IP receptor. Leu172 is both conserved between IP and TP receptors. Gln162 does not lie within close proximity of the opening of the pocket (Figure 5), and the side chain of Met174 is likely not centered enough to make contact with the ligand (Figure 5). Thus in looking at the model, Arg173 becomes the residue with the highest potential for the specific iloprost recognition (Figure 5). Based on this hypothesis, the residue, Arg173, was subjected to mutagenesis studies. First, the Arg residue in the IP eLP_2 _was replaced with Ala to eliminate the charged side chain. After transfection of the mutated IP receptor cDNA into COS-7 cells, a similar expression level of the mutant and wild type IP receptors was confirmed by Western blot (Figure 6A). The binding of the recombinant receptor to its ligand was then performed using [^3^H]-iloprost and unlabeled (cold) iloprost was used as a competitive ligand (Figure 6B). The mutant with the single replacement of the Arg residue with Ala lost its binding activity to the receptor agonist as compared with the wild type (Figure 6B). These data indicate that the Arg residue, as predicted from the IP model, is indeed important in ligand recognition. The results also confirm that the model is indeed useful in providing information for structure and function relationship studies of the IP receptor.

**Figure 7:**
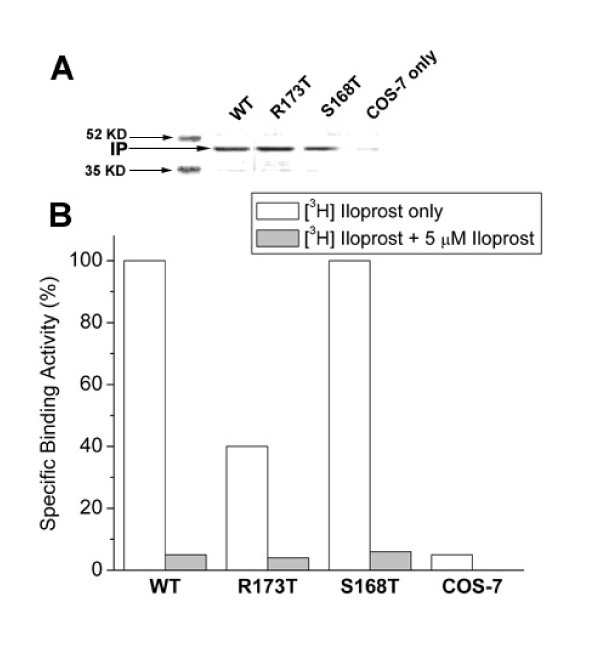
Analysis of the mutation of Arg173 to Thr residue and Ser168 to Thr residue of the recombinant IP receptors. A). Western blot. B). Ligand binding activity. The methods are described in the Figure 6.

**Figure 8:**
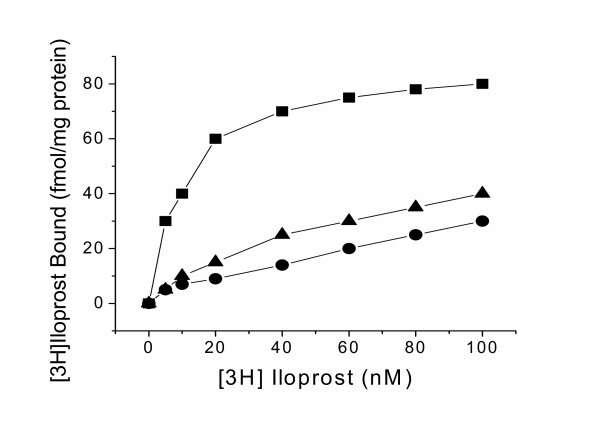
Kinetic properties of [^3^H] iloprost binding to the recombinant IP receptors expressed in COS-7 cells. The cell membrane protein prepared from COS-7 cells that transiently expressed the wild-type (squares), R173A mutant (circles) or R173T mutant (triangles) of the IP receptor was incubated with the increasing concentration of the [^3^H] iloprost.

To further identify the specificity of the Arg residue in the IP receptor, the Arg173 was then mutated to Thr that lies in the corresponding position of the TP receptor (Figure 1). After expression of the mutant in COS-7 cells, confirmed by Western blot (Figure 7A) as described above, the binding of the mutated IP receptor to iloprost was tested. The Arg173Thr mutant retained about 40% ligand binding activity as compared with the wild type IP receptor (Figure 7B). In contrast, the control mutant of the IP receptor, Ser168Thr, which is highly conserved in the all of the prostanoid receptors, retained full activity binding to iloprost (Figure 7B). The impairment of the Arg173 mutants binding to iloprost was further concluded by kinetic studies as shown in Figure 8. These results indicate that Arg173 is specifically important for ligand recognition in the IP receptor. The Arg residue in the IP and Thr residue in the TP may be involved in the determination of their ligand selectivities. If so, this will provide important clues for further characterizing ligand selectivity of other prostanoid receptors using the modeling and mutagenesis approaches.

## Discussion

The lack of the 3-D structural models of the prostanoid receptors has become a major obstacle in both further understanding their molecular mechanisms and in the design of pharmacological intervention strategies. Thus, developing useful approaches for further structural and functional characterization of the GPCRs is crucial. The crystal structure of rhodopsin offers a structural template for the conserved TM helices of other GPCRs, including the prostanoid receptors. The TM domain modeling for the IP and TP receptors, using the x-ray structures of the TM domains of bRH described in the paper is also suitable for the modeling of the TM domains of other prostanoid receptors. However, as described above, the bRH crystal structure provides few structural and functional clues for the extracellular and intracellular domains of the prostanoid receptors. One useful way to characterize the GPCR functions is to assemble information obtained from studies using receptor fragments. Synthetic peptides have been used as important tools in mimicking the functional domains of GPCRs. Peptides corresponding to the C-terminal extramembrane domains of the angiotensin II AT1A receptor [46], natriuretic peptide receptor C [47], testicular follicle stimulating hormone receptor [48] and BR [49,50] have functional activities, which indicate that these peptides can adopt similar structures in the cognate parts of the intact receptors. The synthetic peptides corresponding to the intracellular domains of the M4 subtype muscarinic, cholinergic and α 2-adrenergic receptors could directly bind to and activate their specific G proteins [51]. Also, Yeagle et al have used the NMR structures of the synthetic peptides getting 3D structural information for the intracellular loops of bRH before its crystal structure was available [52].

However, the synthetic peptide studies, giving only fractional information of the interested proteins, have limited use in detailed structural and functional characterization of the interested proteins. Our group has recently been focusing on developing a link between peptide and protein studies into one system to further enhance our ability to characterize the structure/function relationship of proteins. Integrated high-resolution NMR techniques with synthetic peptide and recombinant protein approaches have led to the development of "computation-guided constrained peptide synthesis" and "NMR-experiment-guided mutagenesis" for aiding in the structural and functional characterization of the TP receptor. Interestingly, protein-modeling using the TP eLP NMR structures of other prostanoid receptors has become a new exploration. It is particularly important since it is not likely that crystal structures will available for any prostanoid receptors in the near future, and the high resolution NMR structural determination for the membrane-bound receptor proteins is unlikely to be solved any time soon, and in addition, the NMR instrumentation necessary for protein structural determination is not available in many labs.

In general, the location of the ligand recognition site of the IP receptor is likely similar to the TP receptor because it is known that all prostanoids have cross-binding activities to their receptors. In addition, it is also supported by the fact of that TP eLP2 important to ligand recognition is highly conserved in IP (Figure 2). The achievement of this paper in regards to the successful modeling of the eLPs of the IP receptor using the NMR structures of the TP eLPs has supported the hypothesis in which NMR structures can be applied to the modeling of other prostanoid receptors and use them as a working model for the prediction of ligand recognition sites in general. It is particularly important to note that the modeling has also been tested by key residue mutagenesis using the recombinant IP receptor protein. The remaining work, including further mapping the residues involved in the ligand recognition in the eLPs for the IP receptor by mutagenesis analysis, is currently under progress in our laboratories.

It shall also be noted that in our previous publication [42], the prediction of the residues, Ala177 and Gln178, in the eLP2 important to ligand recognition was supported by our NMR structural studies using synthetic peptide. These two residues are likely not enough to cover the pocket. In this manuscript the additional residues are predicted from the modeling studies based on the NMR structural model of the extracellular loops of the TP receptor. In combination of the two separated studies, we have more confidences that the IP eLP2 provided major residues to form ligand recognition site in the extracellular domains of the IP receptor. In addition, the model also provides important clues for the molecular mechanisms of the interaction between the ligand and the receptor. Based on the docking model showing in Figure 5, several interactions including the charge contact (between the residue Arg173 and the C1 carboxylate of Iloprost), the hydrophobic contact (between the residues A172/ M174/Leu177 and the side chain of Iloprost), and the hydrogen bond contacts (between the residues Q172/Q178 and the C11-OH/C15-OH of Iloprost) are predicted. Of course, the predictions are needed to be further tested by mutagenesis and structural studies.

Finally, identification of the ligand recognition site on the extracellular domain of the IP receptor has no conflict with the identified residues important to the ligand binding in TM domains for GPCRs. As described in previous studies, we have proposed two stages of ligand binding to a prostanoid receptor in which ligand is specifically recognized by the key residues in the extracellular domains of the receptor first, and then deposited into the receptor TM domains [43]. For instance, the highly conserved Arg residue in the TM VII is important to the ligand binding, but it is not likely to be important in determination of the ligand selectivities for the different prostanoid receptors. Thus, identification of the new residues determined the specific ligand recognition in the extracellular domain is important, and has not controversial with the identified ligand binding residues in the TM domain.

## Conclusion

We have constructed a 3D working model for the human IP receptor by homology modeling using the crystal structure of the bovine rhodopsin TM domains and the NMR peptide structures of the extracellular loops of the TP receptor. The residues in the eLP2 domain involved in forming ligand recognition site were proposed. One of the key residues, Arg173 important to the ligand recognition was predicted from the model and confirmed by mutagenesis. The strategy used for the studies is suitable for modeling and prediction of ligand recognition pockets for other prostanoid receptors.

## Materials and methods

### Materials

COS-7 cells were purchased from ATCC (Manassas, VA). Medium for culturing COS-7 cells was from Invitrogen. [^3^H]-iloprost and iloprost were purchased from Amersham Pharmacia Biotech (Piscataway, NJ). DNA polymerase and *Dpn*I endonuclease were obtained from Stratagene (La Jolla, CA).

### Antibody production

HPLC-purified synthetic peptides corresponding to the three extracellular loops were mixed and coupled to keyhole-limpet haemocyanin (KLH) using glutaldehyde. The peptide antibody was produced in Female New Zealand White rabbits by Research Genetic Inc. The specific peptide antibody was obtained by affinity chromatography using the appropriate peptide immobilized on CNBr-activated Sepharose 4B.

### X-ray crystal structure of bRH

The recently reported X-ray crystal structure of bRH with a resolution at 2.6 Å (1L9H) [53] was downloaded from Protein Data Bank (PDB), and the 3-D structures of the TM domains were extracted using Insight II software on a SIG workstation. These structures were used for homology modeling of the TM domains of the IP and TP receptors.

### NMR structures of the synthetic peptides mimicking the extracellular domains of TP receptors

We have solved all of the NMR structures of the constrained peptides mimicking the three eLPs of human TP receptor [43]. These are saved in our database. The NMR structures were converted to PDB format and then used for the homology modeling of the IP eLP domains using the Insight II software on a SIG workstation.

### NMR structure of iloprost

The 3-D structure of iloprost, the IP receptor agonist, was solved by high-resolution 2-D NMR spectroscopy [42], and the conformation was used for directly docking with the IP receptor.

### Molecular modeling and ligand docking

Molecular modeling, dynamic and ligand docking studies were performed on a Silicon Graphics Octane workstation using the software packages Insight II and Discover [54]. The package includes the software for sequence alignment, secondary structural calculation, hydropath analysis, protein modeling, energy minimization, molecular dynamics, molecular annealing and others.

### Site-directed mutagenesis

**A **pAcSG-IP wild-type cDNA was first subcloned into *Eco*RI/*Xba*I sites of pcDNA3.1(+) expression vector. The IP receptor mutants were then constructed using standard PCR. The procedures included the use of a pcDNA3.1(+) vector containing a wild-type IP receptor cDNA as a template, and two synthetic oligonucleotide primers containing the desired mutation for the reaction. The primers, which were complementary to opposite strands of the template, extended during the temperature cycling of 95°C for 30s, 53°C for 1 min 30s, and 68°C for 13 min for a total of 25 cycles with an additional extension cycle of 68°C for 10 min using *Pfu *DNA polymerase. The mutant products were treated with *Dpn*I endonuclease to digest the parental DNA template and confirmed by DNA sequencing. The plasmids were then prepared using a Midiprep kit (Qiagen) for transfection into COS-7 cells for expression.

### Expression of the recombinant IP receptor in COS-7 cells

COS-7 cells were cultured at 37°C in a humidified 5% CO_2 _atmosphere in high glucose Dulbecco's modified Eagle's medium containing 10% fetal bovine serum, antibiotics and antimycotics. The cells, which were placed on 100-mm dishes at a density of 1.0 × 10^6 ^were cultured overnight and then transfected with 10 μg of purified cDNA of pcDNA3.1(+)/IP wild-type or each mutant by the Lipofectamine method [55], as outlined by the manufacture's instructions (Invitrogen). Approximately 48 hours after transfection, the cells were harvested for further protein purification.

### Western blot analysis

The transfected COS-7 cells were scraped from the plates into ice-cold PBS buffer, pH 7.4, and collected by centrifugation. After washing three times, the pellet was resuspended in a small volume of the same buffer. The protein was separated by 12% polyacrylamide gel electrophoresis under denaturing conditions and then transferred to a nitrocellulose membrane. A band recognized by primary antibodies against the peptides mimicking the human IP eLPs was visualized with horseradish peroxidase substrate as previously described [10].

### Ligand binding assay

Ligand binding assay for the IP receptor was performed using the method as described by [40]. Briefly, the cell membrane (0.1 mg) in binding buffer was incubated with 4 nM [^3^H]-iloprost (30,000 cpm) in a 0.1 ml reaction volume at room temperature for 40 min. The reaction was terminated by the addition of 5 ml of ice-cold washing buffer (0.025 M Tris-HCl, pH 7.4). The unbound ligand was then filtered through a Whatman GF/B glass filter (Whatman) under a vacuum. The radioactivity of the receptor-bound [^3^H]-iloprost remaining on the filter was counted in 10 ml of scintillation cocktail using a Beckman β Counter.

## Abbreviations

IP, prostacyclin (prostaglandin I_2 _(PGI_2_)) receptor; TP, thromboxane A_2 _receptor; GPCR, G protein-coupled receptor; NMR, nuclear magnetic resonance; eLP, extracellular loop; eLP1, the first eLP; eLP2, the second eLP; and eLP3, the third eLP.

## Authors' contributions

CHR carried out the homology modeling and the receptor mutagenesis work. JW participated in the final figure preparations. KHR provided modeling design and drafted the manuscript. All authors read and approved the final version of the manuscript
